# Estimated Use of Prescription Medications Among Individuals Incarcerated in Jails and State Prisons in the US

**DOI:** 10.1001/jamahealthforum.2023.0482

**Published:** 2023-04-14

**Authors:** Jill Curran, Brendan Saloner, Tyler N.A. Winkelman, G. Caleb Alexander

**Affiliations:** 1 Center for Drug Safety and Effectiveness, Johns Hopkins Bloomberg School of Public Health, Baltimore, Maryland; 2 Department of Epidemiology, Johns Hopkins Bloomberg School of Public Health, Baltimore, Maryland; 3 Department of Health Policy and Management, Johns Hopkins Bloomberg School of Public Health, Baltimore, Maryland; 4 Division of General Internal Medicine, Hennepin Healthcare, Minneapolis, Minnesota; 5 Health, Homelessness, and Criminal Justice Lab, Hennepin Healthcare Research Institute, Minneapolis, Minnesota; 6 Division of General Internal Medicine, Johns Hopkins Medicine, Baltimore, Maryland

## Abstract

**Importance:**

Although incarcerated individuals experience higher rates of chronic conditions, little is known regarding the use of prescription medications in jails and prisons in the US.

**Objective:**

To characterize treatment with prescription medications in jails and state prisons relative to noncorrectional settings in the US.

**Design, Setting, and Participants:**

This cross-sectional analysis using 2018 to 2020 data from the National Survey on Drug Use and Health (NSDUH) estimated the prevalence of disease among recently incarcerated and nonincarcerated adults in the US. The study used 2018 to 2020 IQVIA’s National Sales Perspective (NSP) to quantify the distribution of medications to incarcerated and nonincarcerated populations. The NSP provides national dollar and unit sales of prescription medications across multiple distribution channels, including prisons and jails. The study population included incarcerated and nonincarcerated individuals from NSDUH. Seven common chronic conditions were assessed. Data were analyzed in May 2022.

**Exposures:**

Medications being sent to correctional facilities vs all other settings in the US.

**Main Outcomes and Measures:**

The main outcomes were distribution of medications to treat diabetes, asthma, hypertension, hepatitis B and C, human immunodeficiency virus (HIV), depression, and severe mental illness to incarcerated and nonincarcerated populations.

**Results:**

The proportion of pharmaceuticals distributed to jails and state prisons to treat type 2 diabetes (0.15%), asthma (0.15%), hypertension (0.18%), hepatitis B or C (1.68%), HIV (0.73%), depression (0.36%), and severe mental illness (0.48%) was much lower compared with the relative burden of disease among this population. The incarcerated population in state prisons and jails accounted for 0.44% (95% CI, 0.34%-0.56%) of estimated individuals with diabetes, 0.85% (95% CI, 0.67%-1.06%) of individuals with asthma, 0.42% (95% CI, 0.35%-0.51%) of hypertension, 3.13% (95% CI, 2.53%-3.84%) of hepatitis B or C, 2.20% (95% CI, 1.51%-3.19%) of HIV, 1.46% (95% CI, 1.33%-1.59%) of depression, and 1.97% (95% CI, 1.81%-2.14%) of severe mental illness. After adjusting for disease prevalence, the relative disparity was 2.9-fold for diabetes, 5.5-fold for asthma, 2.4-fold for hypertension, 1.9-fold for hepatitis B or C, 3.0-fold for HIV, 4.1-fold for depression, and 4.1-fold for severe mental illness.

**Conclusions and Relevance:**

In this cross-sectional, descriptive study of the distribution of prescription medications for chronic conditions in jails and state prisons, the findings suggest that there may be underuse of pharmacological treatment in correctional facilities relative to the nonincarcerated population. These findings, which require further investigation, may reflect inadequate care in jails and prisons and represent a critical public health issue.

## Key Points

**Question:**

How commonly are medications for chronic conditions used in jails and state prisons compared with community settings in the US?

**Findings:**

In this cross-sectional descriptive study of incarcerated and nonincarcerated populations in the US from 2018 to 2020, use of prescription medications for chronic conditions was consistently lower in jails and state prisons compared with community settings. After adjusting for disease prevalence, the relative disparity was 2.9-fold for diabetes, 5.5-fold for asthma, 2.4-fold for hypertension, 1.9-fold for hepatitis B or C, 3.0-fold for human immunodeficiency virus, 4.1-fold for depression, and 4.1-fold for severe mental illness.

**Meaning:**

This analysis suggests that prescription medications for chronic conditions may be substantially underused in jails and state prisons in the US relative to the nonincarcerated population, after accounting for the differential burden of disease in these settings.

## Teaser

This cross-sectional study evaluates treatment with prescription medications in jails and state prisons relative to noncorrectional settings in the US.

## Introduction

There are nearly 2 million people in prisons and jails in the US on any given day, representing the largest incarcerated population in the world.^1^ Individuals in jails and prisons also have significantly higher rates of chronic conditions and mental illness than the general population.^2,3,4,5,6^ While incarcerated individuals have a constitutional right to health care,^7^ in practice correctional health care programs are often underfunded and understaffed.^8,9,10,11,12,13,14^ For example, in one study using the 2002 Survey of Inmates in Local Jails and the 2004 Survey of Inmates in State and Federal Correctional Facilities, Wilper and colleagues^9^ found that many incarcerated people with serious chronic conditions were not receiving appropriate care. Similarly, in a study focusing on a sample of incarcerated individuals from a large county jail from 1995 to 1996, perception of the accessibility and quality of health care services were extremely low.^8^

In addition to studies examining general health care services, other work has documented shortcomings in medication quality and access in correctional settings.^5,6,12,15,16,17^ In a review of more than 400 lawsuits filed over alleged mistreatment of incarcerated people, reporters found that approximately one-third of suicide attempts among people in jail occurred after staff allegedly failed to provide medications for mental illness.^18^ Conversely, there have been studies demonstrating overprescribing of antipsychotics among incarcerated individuals.^19^

Despite the insights from these studies, they leave several questions unanswered, including the degree to which there are continuing differences in the rates of treatment of common, chronic conditions in jails and prisons as compared with nonincarcerated settings. This is important because prescription medications are an important window into broader health care use: almost half of the US population reported using 1 or more prescription drugs in the past 30 days during 2015 to 2016^20^ and use is even higher for adults with chronic diseases. We used a unique data source to characterize the distribution of medications to treat 7 common chronic conditions in incarcerated and nonincarcerated individuals.

## Methods

### Data Sources

In this cross-sectional study, we used 2018 to 2020 data from IQVIA’s National Sales Perspective (NSP) to quantify the distribution of prescription medications to incarcerated and nonincarcerated populations. The present study’s incarcerated population included individuals in jails and state prisons. The NSP data project national sales based on direct measurements of greater than 90% of all retail and nonretail sales from manufacturers and wholesalers through a variety of channels, including state prisons, county and city jails, and juvenile detention centers.^21^ The NSP data report extended units, which measure the number of single items, such as a bottle or a packet of tablets or capsules contained in a unit or shipping package.

Despite the comprehensiveness of the NSP, we nevertheless undertook several steps in an effort to improve the quality and relevance of this study’s analyses. First, because the primary wholesaler of federal accounts discontinued data contribution to IQVIA in 2012, we excluded the federal prison population from all population estimates. Second, the NSP’s jails and prisons channel primarily represents pharmaceuticals distributed through correctional pharmacies; while not universal, such pharmacies are the dominant contracting model in most prison systems.^14^ We consulted with 4 experts in correctional health care, including medical directors from contracted vendors, who estimated that this channel accounts for at least three-fourths of the pharmaceutical volume. In the main analyses, we conservatively estimated that the NSP channel only accounted for one-half of pharmaceuticals in correctional facilities.

We combined information from IQVIA with 2018 to 2020 data from the National Survey on Drug Use and Health (NSDUH), a nationally representative household survey of individuals aged 12 years and older.^22^ To estimate condition prevalence among the incarcerated population, we analyzed individuals in NSDUH with recent criminal-legal involvement. The NSDUH data include individuals living in noninstitutional group housing (eg, college dormitories, but not jails or prisons) or in temporary housing, such as residence in a shelter. The NSDUH data use multistage area probability sample for each US state and the District of Columbia and computer-assisted personal interviewing with an interviewer present in addition to audio computer-assisted self-interviewing to support confidential responses. We limited the study sample to US adults aged 18 years and older.

The NSDUH does not survey people in jails and prisons, but it did allow us to identify people who were recently incarcerated, specifically individuals currently on parole or have been arrested and booked in the prior year. The nonincarcerated population included individuals without any parole, probation, or arrest in the last year. We used population estimates for state prisons^23^ and local jails^24^ and US census data^25^ to calculate the number of individuals affected in each incarcerated group. Incarcerated population and US census population estimates were averaged over 2018, 2019, and 2020. The NSDUH defines severe mental illness from a model that incorporates the K6 Psychological Distress Scale, World Health Organization Disability Assessment Schedule, thoughts of suicide in the past year, major depressive episode in the past year, and age.

### Selection of Pharmaceutical Classes

We assessed medication use for diabetes, asthma, hypertension, hepatitis, human immunodeficiency virus (HIV), depression, and severe mental illness, as these conditions are commonly treated with prescription medications, could be disaggregated in the NSDUH, and are among the most prevalent diseases among people who are incarcerated. We used IQVIA’s Uniform System of Classification (USC) to identify and group pharmaceutical products relevant to each chronic condition.^26^ In the case of diabetes, we examined whether there was evidence of disproportionate use of older products among incarcerated individuals compared with their counterparts, sorting therapeutics based on: oldest vintage (1920s-1960s: human insulins, sulfonylureas, and biguanides), moderate vintage (1990s: α-glucosidase inhibitors, analogues of human insulins, glinides, glitazones) and newest vintage (2000s-present: antidiabetic hormone analogues, dopamine receptor agonists, dipeptidyl peptidase 4 inhibitors, sodium-glucose cotransporter 2 inhibitors).

### Statistical Analysis

We used descriptive statistics to perform the analysis. We calculated prevalence rates and 95% CIs for both the recently incarcerated and nonincarcerated populations using NSDUH. We used STATA MP, version 15.1 (StataCorp LLC) for all analyses related to NSDUH and accounted for the complex survey design. We multiplied the NSDUH prevalence rates by population estimates from the Bureau of Justice^15,16^ and the US census^17^ to calculate the number of incarcerated and nonincarcerated individuals affected by each condition along with a lower and upper bound based on the NSDUH 95% CIs. We used these numbers to estimate the proportion of disease burden and corresponding 95% CIs among incarcerated individuals. Similarly, we calculated the proportion of pharmaceutical distribution for each condition going to jails and prisons vs the general population. A magnitude of difference was calculated by dividing the percentage of disease among the incarcerated population by the percentage of NSP distribution to jails and prisons. We included a lower and upper bound for these magnitudes of difference using the 95% CIs from the NSDUH prevalence estimates. We conducted a cross-sectional analysis and therefore followed the Strengthening the Reporting of Observational Studies in Epidemiology (STROBE) reporting guideline. The data were exempt from review by an institutional review board at the Johns Hopkins Bloomberg School of Public Health. Informed consent was waived because the data were deidentified. Analyses were conducted in May 2022.

### Sensitivity Analyses

We performed sensitivity analyses to examine how these findings varied based on assumptions regarding the prevalence of the conditions of interest. For the incarcerated population, we used the 2011 to 2012 National Inmate Survey and the 2016 Survey of Prison Inmates, which both consisted of in-person surveys of individuals in correctional facilities; these estimates were derived from published reports that accounted for the complex sampling schemes of the surveys.^27,28,29,30^ We used multiple sources to identify 2016 estimates of these conditions in the nonincarcerated population including the National Health Interview Study, the Centers for Disease Control and Prevention, the National Health and Nutrition Examination Survey, and the National Institute of Mental Health.^31,32,33,34^ Using these prevalence rates, the sensitivity analyses were conducted using the same methods as the primary analysis. Because these were different data sources, hepatitis for sensitivity analyses only included hepatitis C and bipolar affective disorder was used instead of severe mental illness.

## Results

### Prevalence of Chronic Conditions

Of the conditions that were evaluated, depression had the highest prevalence among the incarcerated population at 15.10% (95% CI, 13.36%-17.01%). In the general population, hypertension was the most common of the conditions assessed at 25.29%, (95% CI, 24.73%-25.85%), followed by diabetes at 13.88% (95% CI, 13.44%-14.33%) (Figure). The prevalence of hepatitis at 6.08% (95% CI, 4.32%-8.49%) vs 1.41% (95% CI, 1.25%-1.59%), HIV at 0.84% (95% CI, 0.43%-1.61%) vs 0.28% (95% CI, 0.21%-0.37%), depression at 15.10% (95% CI, 13.36%-17.01%) vs 7.64% (95% CI, 7.40%-7.88%) and severe mental illness at 13.12% (95% CI, 11.59%-14.82%) vs 4.89% (95% CI, 4.70%-5.08%) were noticeably higher in state prisons and jails compared with the general population. The estimated total number of incarcerated and nonincarcerated individuals affected by each condition along with 95% CIs are represented in Table 1.

**Figure.**
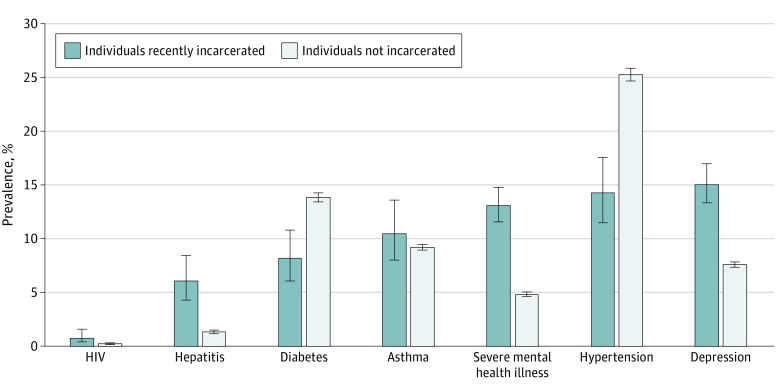
Prevalence Rates of Select Conditions Among Recently Incarcerated and Nonincarcerated Individuals in the US Sources: 2018-2020 National Survey on Drug Use and Health,^21^ the US Census Bureau.^24^ Error bars represent 95% CIs.

**Table 1.** Estimated Number of Individuals With Select Chronic and Mental Health Conditions Among Recently Incarcerated and Nonincarcerated Individuals in the US^a^

Condition	No. (95% CI)	
	Recently incarcerated	Nonincarcerated
Diabetes	153.94 (115.56-203.63)	34 786.70 (33 683.96-35 914.51)
Asthma	197.44 (151.09-255.76)	23 167.74 (22 440.93-23 912.10)
Hypertension	268.51 (216.76-330.20)	63 382.98 (61 979.48-64 786.48)
Hepatitis B or C	114.00 (81.04-159.17)	3526.29 (3120.28-3984.93)
HIV	15.66 (8.09-30.21)	695.73 (522.82-915.53)
Depression	283.14 (250.51-318.95)	19 147.72 (18 553.74-19 756.74)
Severe mental illness	246.01 (217.32-277.88)	12 245.52 (11 784.37-12 721.71)

Abbreviation: HIV, human immunodeficiency virus.

^a^ Sources: 2018-2020 National Survey on Drug Use and Health,^21^ the US Census Bureau. ^24^ Values reported in thousands.

### Diabetes Pharmaceutical Volume in Incarcerated and Nonincarcerated Populations

The incarcerated population accounted for 0.44% (95 % CI, 0.34%-0.56%) of the diabetes disease burden. However, 0.15% of oral antihyperglyemic pharmaceutical volume from 2018 to 2020 was distributed to jails and state prisons. This represents a 2.86-fold (95% CI, 2.22-3.65) difference between diabetes disease burden and medication treatment in jails and state prisons (Table 2). There was a marked difference in the distribution of diabetes therapies of varying vintages in jails and state prisons as compared with other settings; among the incarcerated, of all diabetes therapies, 87.1% were of the oldest vintage, 11.1% were of moderate vintage, and 1.8% were of the most recent vintage. By contrast, among the nonincarcerated population, 76.5% of diabetes therapies were of the oldest vintage, 11.2% were of moderate vintage, and 12.3% were of the most recent vintage.

**Table 2.** Magnitude of Difference Between the Burden of Disease Among the Recently Incarcerated vs Proportion of Total National Sales Perspective (NSP) Extended Units Distributed to the Incarcerated Population^a^

	Disease among recently incarcerated population, % (95% CI)	Total NSP to incarcerated population, %	Magnitude of difference (95% CI)^b^
Diabetes	0.44 (0.34-0.56)	0.15	2.86 (2.22-3.65)
Asthma	0.85 (0.67-1.06)	0.15	5.45 (4.32-6.83)
Hypertension	0.42 (0.35-0.51)	0.18	2.40 (1.98-2.89)
Hepatitis B or C	3.13 (2.53-3.84)	1.68	1.87 (1.51-2.29)
HIV	2.20 (1.51-3.19)	0.73	3.01 (2.06-4.37)
Depression	1.46 (1.33-1.59)	0.36	4.08 (3.73-4.45)
Severe mental illness	1.97 (1.81-2.14)	0.48	4.11 (3.78-4.46)

Abbreviation: HIV, human immunodeficiency virus.

^a^ Sources: 2018-2020 National Survey on Drug Use and Health,^21^ the US Census Bureau,^24^ IQVIA’s National Sales Perspective.^19^ For asthma, Uniform System of Classification codes associated with asthma treatment under 28000 (respiratory treatment) were used. Hypertension: 41200 (diuretics), 31410 (β-blockers), 31110 (angiotensin-converting enzyme inhibitors), 31120 (angiotensin II antagonists), 31300 (calcium blockers), 31440 (α blockers), 31420 (α-β blockers), and 31450 (central-acting agents). Hepatitis: 82212 (hepatitis C antivirals), 86210 (immunologic interferons), and 82211 (hepatitis B antivirals). HIV: 82100 (HIV antivirals). Depression: 64310 (tricyclics and tetracyclics), 64320 (monoamine oxidase inhibitors), 64340 (selective serotonin reuptake inhibitors), 64350 (serotonin and noradrenaline reuptake inhibitors), and 64330 (other antidepressants). Severe mental illness: 64100 (antipsychotics) and 64400 (mood stabilizers).

^b^ The magnitude of difference represents a relative ratio. Dividing the first 2 columns will not equal the third column exactly due to rounding errors.

### Distribution of Pharmaceuticals for Other Conditions

The disparity between burden of disease and pharmaceutical volume in incarcerated and nonincarcerated populations was also present for asthma, hypertension, hepatitis, HIV, depression and severe mental illness (Table 2). For example, the incarcerated population accounted for 0.85% (95% CI, 0.67%-1.06%) of individuals with asthma, but 0.15% of asthma treatment volume was distributed to jails and state prisons, a 5.45-fold (95% CI, 4.32-6.83) relative difference. For HIV, 2.20% (95% CI, 1.51%-3.19%) of the disease population was among incarcerated individuals, but 0.73% of treatment volume was to the incarcerated population, a 3.01-fold (95% CI, 2.06-4.37) difference.

The proportion of pharmaceuticals distributed to jails and state prisons to treat hypertension (0.18%), hepatitis B or C (1.68%), depression (0.36%), and severe mental illness (0.48%) was much lower compared with the relative burden of disease among this population. The incarcerated population in state prisons and jails accounted for 0.42% (95% CI, 0.35%-0.51%) of individuals with hypertension, 3.13% (95% CI, 2.53%-3.84%) of individuals with hepatitis B or C, 1.46% (95% CI, 1.33%-1.59%) of individuals with depression, and 1.97% (95% CI, 1.81%-2.14%) of individuals with severe mental illness. After adjusting for disease prevalence, the relative disparity was 2.4-fold for hypertension, 1.9-fold for hepatitis B or C, 4.1-fold for depression, and 4.1-fold for severe mental illness.

### Sensitivity Analyses

Results from the sensitivity analyses yielded substantively similar conclusions as the primary analyses. Prevalence rates were higher among the incarcerated populations for most conditions compared with the nonincarcerated population (eTable 1 in Supplement 1). However, the prevalence rates among the incarcerated population for hypertension, depression, and bipolar affective disorder were significantly higher in the sensitivity analyses compared with the primary analyses. In the sensitivity analyses, we observed a greater disparity between disease burden and pharmaceutical volume in incarcerated and nonincarcerated populations for each condition other than HIV (eTable 2 in Supplement 1). Magnitude of differences ranged from 2.37 for HIV up to 10.63 for bipolar affective disorder.

## Discussion

In this cross-sectional, descriptive analysis combining recent information from a large federal survey with data on national prescription drug distribution, the proportion of disease among the recently incarcerated population was substantially greater than the proportion of total pharmaceutical volume assessed allocated to that population for each condition that was evaluated. The relative disparity between disease burden and pharmaceutical volume varied from 1.9-fold to 5.5-fold and was greatest for asthma and least for hepatitis. While many deficiencies in the correctional health care system have been well described,^2,35^ to our knowledge, the present study is the first to use national data to document substantial disparities in receipt of medications for chronic conditions among people who are incarcerated. These findings, if confirmed in further evaluations, suggest that improved oversight from state and local authorities are needed to ensure adequate pharmacologic treatment of individuals incarcerated within US jails and prisons.^36^

These findings may help to explain some of the disparities in health outcomes that are experienced by those who have been previously incarcerated, and they point to opportunities to intervene upstream to preempt such outcomes. Each year approximately 600 000 people are released from federal and state prisons with another 9 million passing through local jails.^37^ People recently released from prisons tend to experience poorly controlled disease, worse health-related outcomes, and mortality.^38^ Improvement in care and treatment while incarcerated could potentially help mitigate some of the health disparities among people with criminal-legal involvement and thus mitigate some of the harms of mass incarceration.

The present analyses may reflect a variety of different systemic and clinical factors. First, there is widespread underdiagnosis of many of the chronic conditions that were examined,^39,40^ and the rates of such underdiagnosis may be greater among individuals more likely to intersect with the criminal-legal system, including members of racial and ethnic minority groups as well as those of lower socioeconomic status.^41^ Improving the identification and treatment of cardiovascular diseases such as hypertension in correctional facilities may help to reduce wide and well-described racial and ethnic disparities in cardiovascular outcomes. Second, and relatedly, pharmacologic care for conditions such as diabetes and depression is predicated on a regular source of care. Incarcerated people often lack a regular source of care in the community, which can be a further source of delayed care during incarceration. This is especially true in jails, where the length of stay is often just a few days and where the facility may be unlikely to manage a chronic condition that is not already treated in the community. Third, patients in correctional facilities often lack trust in their clinicians and are therefore hesitant to start medications, especially when it is uncertain whether they will be able to continue treatment after release. Although many people who are incarcerated are eligible for Medicaid when they return to the community, restarting treatment is often delayed as they reenroll and identify clinicians.^42,43,44,45^ Finally, treatments for some of the conditions that were examined, such as direct-acting antiviral treatments for hepatitis C, are often quite expensive, and the high cost of new antiviral treatments may be unsustainable by the prison system, as was shown in the case of sofosbuvir.^46^ We found the least discrepancy between disease burden and medication volume for hepatitis C, which may partially be attributed to the new steps states are taking in negotiating the price of treatment for hepatitis C.^47^ The large discordance that we identified in the use of newer treatments for type 2 diabetes is consistent with the economics of these therapies, which may contribute to their underuse in correctional settings. However, without knowing the severity of disease in each setting, it is difficult to interpret these data, as incarcerated individuals with diabetes may be new diagnoses and thus require first-line treatment. Even for more common chronic disease medications, there may be access issues because of the limited staffing present in many facilities, including physicians qualified to diagnose and manage medications, whether or not the cost of the medicines themselves are prohibitive.

Several of the conditions that we examine have particular relevance when considering the health of the incarcerated population. For example, failure to identify and comprehensively treat viral infections such as HIV and hepatitis B and C in correctional settings represents a missed opportunity to treat and prevent infections in US jails and prisons.^48^ Similarly, research demonstrates higher rates of psychiatric disorders among the incarcerated than general population, and such individuals are at elevated risk of suicide, self-harm, violence, and victimization.^49^ While data from California suggest that psychotropic medication use has increased proportionate with the burden of mental illness in carceral facilities, there is a lack of national data on trends in prevalence and medication use.^50^ Targeted initiatives, including additional oversight from health care agencies, to further characterize and abate any undertreatment of mental health disorders among this population are needed.^51^

### Limitations

First, the estimates of the prevalence of different diseases were based on cross-sectional, self-reported survey data. However, a strength of NSDUH, in contrast to the National Inmate Survey and the 2016 Survey of Prison Inmates, which we used in sensitivity analyses, is that we were able to estimate chronic condition prevalence for recently incarcerated and nonincarcerated populations within the same data source while also deriving CIs for these estimates. Others have called for a dedicated national survey of the health and health care of individuals within correctional facilities in the US, and such an ongoing assessment would be useful to further evaluate these findings.^52,53,54^ Second, while IQVIA’s NSP is the most comprehensive source of information about prescription drug distribution to jails and prisons that we are aware of, it does not represent a complete census of these channels. Specifically, NSP lacks data on prescriptions not filled by correctional pharmacies, such as those received through the 340B program. Several departments of correction enter into these agreements specifically to cover expensive treatments, such as those for hepatitis C and HIV and acquired immunodeficiency syndrome.^13^ To account for this, we conservatively doubled the correctional prescription data in the primary estimates, assuming NSP only accounts for one-half of pharmaceuticals distributed to jails and state prisons. However, these estimates are still bound by uncertainty because of the nature of the pharmaceutical supply chain to jails and prisons. We were unable to exclude prescriptions to juveniles from the estimates, even though we focused on disease prevalence in adult populations. Additionally, we were unable to account for population differences in potential covariates, such as age. Higher average age among the nonincarcerated population leads to a higher prevalence of certain conditions, such as diabetes, which would imply a higher magnitude of medications prescribed to the nonincarcerated population. However, we aimed to assess the prescription volume relative to prevalence estimates in order to account for different magnitudes of disease within each population. We also were unable to account for prescription dose in the present analyses, because we measured volume based on extended units. Finally, the present analysis was not designed to assess appropriateness of care received in either correctional or noncorrectional settings.

These findings highlight the importance of measuring quality of care among people with chronic conditions in jails and prisons. Further research is needed to examine trends over time, in addition to regional differences in prisons vs jails, which would help validate these novel findings. We were unable to examine medication use among the federal prison population because we did not have access to prescription volume in federal prisons in our data set. More recent surveys of people in jails and prisons, including direct measures of the medication they receive for chronic conditions, could improve pharmaceutical access and health outcomes in this population. These findings, while limited based on available data, provide a first step as one of the only national level perspectives on pharmacologic treatment among incarcerated individuals compared with the general population. The limitations included highlight the lack of high-quality data available to study this important public health question.^55,56^

## Conclusions

Although people who are incarcerated have a constitutional right to health care, in this cross-sectional, descriptive study assessing prescription volume relative to disease burden, we found evidence suggesting substantially lower use of medications for chronic conditions in jails and state prisons compared with the nonincarcerated population. To our knowledge, this is the first study to characterize these differences in a national population. These findings may represent a substantial opportunity to improve the health of individuals incarcerated in the US.

## Notes

Supplement 1. **eTable 1.** Estimated prevalence of select chronic and mental health conditions among incarcerated and nonincarcerated individuals in the United States (values reported in thousands) using alternative sources to estimate disease prevalence **eTable 2.** Magnitude of difference between the burden of disease among the incarcerated versus proportion of total National Sales Perspective (NSP) extended units distributed to the incarcerated population using alternative sources to estimate disease prevalence

Supplement 2.
Data sharing statement
