# Supplementary material for: Estimated Use of Prescription Medications Among Individuals Incarcerated in Jails and State Prisons in the US
Source: JAMA Health Forum. 2023 Apr 14;4(4):e230482. doi: 10.1001/jamahealthforum.2023.0482 (PMC10105311; doi:10.1001/jamahealthforum.2023.0482)
Supplement: Supplement 1. — eTable 1. Estimated prevalence of select chronic and mental health conditions among incarcerated and nonincarcerated individuals in the United States (values reported in thousands) using alternative sources to estimate disease prevalence eTable 2. Magnitude of difference between the burden of disease among the incarcerated versus proportion of total National Sales Perspective (NSP) extended units distributed to the incarcerated population using alternative sources to estimate disease prevalence [file jamahealthforum-e230482-s001.pdf]

Supplemental Online Content

Curran J, Saloner B, Winkelman TNA, Alexander GC. Estimated use of prescription medications among individuals incarcerated in jails and state prisons in the US. *JAMA Health Forum.* 2023;4(4):e230482. doi:10.1001/jamahealthforum.2023.0482

eTable 1. Estimated prevalence of select chronic and mental health conditions among incarcerated and nonincarcerated individuals in the United States (values reported in thousands) using alternative sources to estimate disease prevalence

eTable 2. Magnitude of difference between the burden of disease among the incarcerated versus proportion of total National Sales Perspective (NSP) extended units distributed to the incarcerated population using alternative sources to estimate disease prevalence

This supplemental material has been provided by the authors to give readers additional information about their work.

© 2023 Curran J et al. *JAMA Health Forum.*

eTable 1. Estimated prevalence of select chronic and mental health conditions among incarcerated and nonincarcerated individuals in the United States (values reported in thousands) using alternative sources to estimate disease prevalence (see footnote).

Condition	Incarcerated, N (%)	Non-Incarcerated (US), N (%)
Diabetes	124.10 (6.2)	21,543.02 (8.8)
Asthma	263.48 (13.1)	20,318.98 (8.3)
Hypertension	432.74 (21.4)	60,956.94 (24.9)
Hepatitis C	139.38 (6.9)	2,129.82 (0.9)
HIV	23.60 (1.2)	1,125.00 (0.5)
Depression	571.50 (28.3)	19,196.11 (8.1)
Bipolar Affective Disorder	481.49 (23.9)	6,854.60 (2.8)

Sources: 2011-12 National Inmate Survey, 2016 Survey of Prison Inmates, National Health Interview Study (NHIS), the Centers for Disease Control and Prevention (CDC), the National Health and Nutrition Examination Survey (NHANES), the National Institute of Mental Health (NIMH), the U.S. Census Bureau

© 2023 Curran J et al. *JAMA Health Forum.*

eTable 2. Magnitude of difference between the burden of disease among the incarcerated versus proportion of total National Sales Perspective (NSP) extended units distributed to the incarcerated population using alternative sources to estimate disease prevalence (see footnote).

	Percent of disease among incarcerated population	Percent of total NSP to incarcerated population	Magnitude of difference
Diabetes	0.57%	0.17%	3.4
Asthma	1.28%	0.16%	8.0
Hypertension	0.70%	0.21%	3.3
Hepatitis C	6.14%	2.08%	3.0
HIV	2.05%	0.87%	2.4
Depression	2.89%	0.45%	6.4
Bipolar Affective Disorder	6.56%	0.62%	10.6

Sources: 2011-12 National Inmate Survey, 2016 Survey of Prison Inmates, National Health Interview Study (NHIS), the Centers for Disease Control and Prevention (CDC), the National Health and Nutrition Examination Survey (NHANES), the National Institute of Mental Health (NIMH), the U.S. Census Bureau, IQVIA's National Sales Perspective

© 2023 Curran J et al. *JAMA Health Forum.*
