# Supplementary material for: Estimated Use of Prescription Medications Among Individuals Incarcerated in Jails and State Prisons in the US
Source: JAMA Health Forum. 2023 Apr 14;4(4):e230482. doi: 10.1001/jamahealthforum.2023.0482 (PMC10105311; doi:10.1001/jamahealthforum.2023.0482)
Supplement: Supplement 2. — Data sharing statement [file jamahealthforum-e230482-s002.pdf]

Data Sharing Statement

Curran. Estimated Use of Prescription Medications Among Individuals Incarcerated in Jails and State Prisons in the US. *JAMA Health Forum*. Published April 14, 2023.
doi:10.1001/jamahealthforum.2023.0482

Data
Data available: No

Additional Information
Explanation for why data not available: Analyses were based on publicly available data as well as data from IQVIA that was accessed under license. De-identified study data reflecting the publicly available data accessed will be made available upon request following publication and ending three years following article publication by request to the corresponding author and at the discretion of the research team.
